# Fibrinolytic Changes in Critical Illnesses: Is Fibrinolysis Shutdown a Specific Concept?

**DOI:** 10.14789/ejmj.JMJ24-0035-P

**Published:** 2024-12-31

**Authors:** JERROLD H. LEVY, TOSHIAKI IBA

**Affiliations:** 1 Department of Anesthesiology, Critical Care, and Surgery, Duke University School of Medicine, Durham, NC, USA; 1 Department of Anesthesiology, Critical Care, and Surgery, Duke University School of Medicine, Durham, NC, USA; 2 Department of Emergency and Disaster Medicine, Juntendo University Graduate School of Medicine, Tokyo, Japan; 2 Department of Emergency and Disaster Medicine, Juntendo University Graduate School of Medicine, Tokyo, Japan

**Keywords:** fibrinolysis, trauma, sepsis, hemostasis, coagulopathy

## Abstract

Trauma-induced coagulopathy (TIC) is characterized by dynamic changes in fibrinolysis, which can significantly impact patient outcomes. These changes typically manifest in two phases: hyperfibrinolysis followed by fibrinolysis suppression. In the early stages of TIC, there is often an overwhelming release of tissue plasminogen activator, which leads to excessive fibrinolysis. This hyperfibrinolytic state results in rapid clot breakdown, leading to uncontrolled bleeding and increased mortality. Following the hyperfibrinolytic phase, the fibrinolysis system is suppressed rapidly due to the increased production of plasminogen activator inhibitor-1, leading to fibrinolysis shutdown. This is a state where clot breakdown is significantly reduced, which can contribute to thromboembolic complications and multi-organ failure. Tranexamic acid, a plasmin inhibitor, effectively regulates hyperfibrinolysis as long as it is used in the appropriate hyperfibrinolytic phase. In summary, TIC involves a complex interplay between hyperfibrinolysis and fibrinolysis shutdown, with the balance between these states being crucial for patient survival. Effective management of TIC requires an understanding of these dynamic changes to tailor therapeutic interventions appropriately.

## Introduction

Sepsis was once defined as a systemic inflammatory response syndrome (SIRS) caused by infection, and it became widely recognized that coagulation and inflammation activation contributed to adverse outcomes. Although the definition of sepsis has since changed to describe a severe infection accompanied by organ dysfunction, the concept that inflammation and coagulation contribute to disease severity remains unchanged. In this setting, coagulation activation ranges from mild coagulation abnormalities to disseminated intravascular coagulation (DIC), along with fibrinolysis due to the interplay of thromboinflammation, a pathological occurring in sepsis and other conditions of injury such as major trauma and heatstroke^1)^. Further, activation of coagulation with decreased anticoagulant activity caused by inflammation and tissue damage contributes to systemic microvascular thrombosis, playing a critical role in multiorgan dysfunction development. Fibrinolytic responses vary extensively from activation to relative suppression often termed fibrinolytic shutdown. This variability in responses influences the clinical manifestations of either thrombotic or hemorrhagic tendencies^2)^.

The activity of plasmin, which regulates fibrinolytic function, is controlled by the balance between tissue-type plasminogen activator (tPA) and plasminogen activator inhibitor 1 (PAI-1), although there are differences in the production, storage, and release patterns of these two factors. tPA is a serine protease primarily produced by endothelial cells and stored in the Weibel-Palade bodies within these cells, whereas PAI-1 is a glycoprotein produced by endothelial cells, adipocytes, and megakaryocytes and subsequently stored in platelets. During vascular injury, stored tPA is rapidly released into the bloodstream via exocytosis triggered by thrombin stimulation, while PAI-1 production increases after the insult due to induced protein synthesis, leading to a significant rise in plasma levels up to 100 times higher. These differences in the dynamics of tPA and PAI-1 are believed to result in the dynamic changes in fibrinolytic function observed under such stress. For example, in cases of severe trauma, the explosive increase in thrombin production (thrombin burst) in response to tissue damage triggers a transient release of stored tPA, leading to fibrinolytic activation. The rapid increase of fibrinolysis, coupled with the loss and consumption of coagulation factors, results in an impaired hemostasis, causing a hemorrhagic tendency that poses a significant challenge for trauma surgeons in achieving hemostasis^3)^.

The effectiveness of antifibrinolytic therapy in response to such fibrinolytic activation has been extensively studied. The CRASH-2 trial demonstrated that administering tranexamic acid within three hours of injury but optimally within one hour significantly reduced in-hospital mortality^4)^. Similarly, the CRASH-3 trial reported that the same treatment improved mortality rates and outcomes associated with traumatic brain injury^5)^. It is crucial to note that for antifibrinolytic therapy to be effective, tranexamic acid must be administered within the appropriate time window. If administered outside this window, the treatment is ineffective and may also worsen the outcomes. This time-sensitive administration of tranexamic acid is a fundamental principle in antifibrinolytic therapy for trauma.

**Figure 1:**
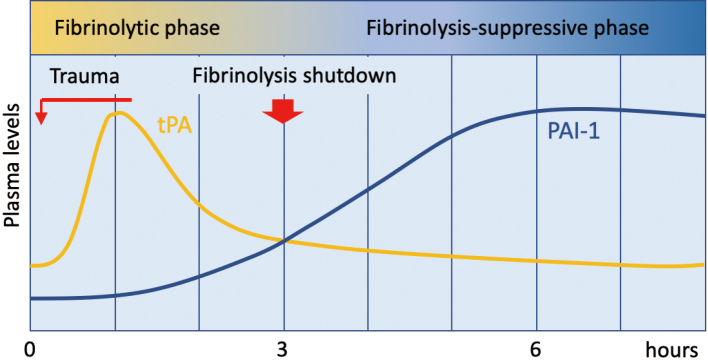
Changes in fibrinolytic activity after trauma Tissue plasminogen activator (tPA) is released immediately after trauma, triggered by a thrombin burst, followed by continuous production of plasminogen activator inhibitor-1 (PAI-1). PAI-1 neutralizes tPA by binding in a 1:1 ratio, suppressing fibrinolytic activity. Approximately three hours after the trauma, fibrinolysis is followed by inhibition of fibrinolysis, a phase known as fibrinolytic shutdown.

Following trauma, sustained production of PAI-1 is induced as a counteraction to the initial hyperfibrinolytic state, leading to a subsequent hypofibrinolytic state. This dynamic shift from hyperfibrinolysis to hypofibrinolysis, known as fibrinolytic shutdown, initially gained attention in trauma surgery (Figure 1). Regarding the temporal changes in fibrinolytic function following trauma, Nakae et al.^6)^ conducted a study on 61 cases of traumatic brain injury who were transported within one hour of injury. They monitored coagulation and fibrinolytic functions over time and reported that hypercoagulation, assessed by thrombin-antithrombin complex (TAT), peaked immediately after injury, while fibrinolytic activity, indicated by D-dimer levels, peaked three hours post-injury. In contrast, PAI-1 levels peaked six hours after injury. These changes in coagulation and fibrinolytic functions were more pronounced in patients with poor long-term outcomes. Additionally, they identified that D- dimer levels within three hours of injury and PAI-1 levels from six hours to the following day were biomarkers associated with long-term prognosis.

The term “fibrinolytic shutdown” suggests a complete cessation of fibrinolytic function; however, it refers to a state of relative fibrinolytic suppression in response to the activated coagulation rather than a complete suppression of fibrinolytic activity. This fibrinolytic suppression, or hypofibrinolysis, is also commonly observed during other forms of physiological stress, such as in sepsis or COVID-19, and can be considered a form of fibrinolytic suppression-type DIC in a broader sense^7)^. While relative fibrinolytic suppression is widely recognized as a driving force toward multiple organ dysfunction, unlike in traumatic coagulopathy, the preceding hyperfibrinolytic state is not often a clinical issue in these conditions. This is partly because it is difficult to pinpoint the onset of infection in sepsis, making it challenging to clinically observe the hyperfibrinolytic state associated with tPA release. Therefore, considering the context in which the term “fibrinolytic shutdown” was originally proposed, its use may not be entirely appropriate in sepsis or similar conditions. The differences in the understanding and use of these terms have long fueled debates between trauma surgeons and intensivists regarding the similarities between trauma-induced coagulopathy (TIC) and DIC. From the perspective of trauma surgeons, Moore et al.^8)^ have advocated for distinguishing between fibrinolytic shutdown in trauma and the low fibrinolytic activity observed in sepsis. This distinction is also relevant to the fibrinolytic suppression commonly discussed in COVID-19^9)^.

In the case of infections, fibrinolytic suppression can be viewed as part of the host defense mechanism, as it aids in trapping pathogens within fibrin, contributing to infection control. However, this suppression also impedes the dissolution of microthrombi, potentially leading to multiple organ failure. With advances in infection management, regulating fibrinolysis might emerge as a novel therapeutic strategy in the modern era. As mentioned earlier, blood PAI-1 levels exhibit significant fluctuations and are not merely transient, making them a promising biomarker for reflecting the severity of illness. This is likely because PAI-1 production is stimulated by inflammatory cytokines such as interleukin- 1 (IL-1) and transforming growth factor-β (TGF-β), as well as by transcription factors like hypoxia- inducible factor-1 (HIF-1), which respond to hypoxic conditions. It is important to note that current PAI- 1 measurement kits assess the tPA/PAI-1 complex (total PAI-1) rather than PAI-1 itself.

One of the recent advancements in the assessment of fibrinolytic function is the widespread use of viscoelastic tests (VET), such as thromboelastography (TEG) and rotational thromboelastometry (ROTEM). Traditionally, fibrinolytic function has been evaluated by measuring biomarkers such as D-dimer, plasmin-α2 plasmin inhibitor complex (PIC), and PAI-1. However, VET is increasingly used in the evaluation of hemostatic and fibrinolytic abnormalities in trauma and cardiovascular surgery. The utility of VET lies in its ability to provide rapid results as a point-of-care test (POCT), making it particularly useful in these critical settings. Fibrinolytic hyperactivity is a significant factor contributing to difficulties in achieving hemostasis, and studies have shown that fibrinolytic hyperactivity detected by VET is associated with the need for transfusions and patient outcomes^10)^. However, there are conflicting reports on this association, and the utility of VET in assessing relative fibrinolytic suppression, such as in sepsis, remains unclear and requires further investigation. The evaluation of fibrinolytic function under various types of stress will need to continue, with additional studies focusing on specific underlying conditions.

## Funding

No funding was received.

## Author contributions

JHL and TI wrote and reviewed the manuscript. Both authors read and approved the final manuscript.

## Conflicts of interest statement

The authors declare that they have no conflict of interest. Toshiaki Iba, one of the Editorial Board members of JMJ was not involved in the peer review or decision-making process for this paper.
